# Artificial intelligence-driven virtual reality eye-tracking for the objective measurement of MRD1 and MRD2 in blepharoptosis

**DOI:** 10.1038/s41598-026-48931-3

**Published:** 2026-04-14

**Authors:** Reenette Savant, Swati Parida, Callum Hunt, Irem Karaer, Mario Frank Farrugia, Agni Mokka, Steven Isherwood, Vanessa Rodwell, Gail D. E. Maconachie, Seema Teli, Michael Hisaund, Zhanhan Tu, Antonella Berry-Brincat, Ian De Silva, Joyce Burns, Mary Awad, Raghavan Sampath, Mervyn G. Thomas

**Affiliations:** 1 Ulverscroft Eye Unit, School of Psychology and Vision Sciences, University of Leicester, Leicester, LE2 7LX UK; 2 Department of Ophthalmology, Leicester Royal Infirmary, University Hospitals of Leicester NHS Trust, Infirmary Square, Leicester, LE1 5WW UK; 3 School of Allied Health Professions, Pharmacy, Nursing and Midwifery, Faculty of Health, University of Sheffield, Sheffield, S10 2TS UK

**Keywords:** Blepharoptosis, Artificial intelligence, Ptosis, Virtual reality, Feasibility, Reproducibility, Computational biology and bioinformatics, Diseases, Engineering, Health care, Mathematics and computing, Medical research

## Abstract

Accurate measurement of eyelid position is essential for diagnosis and surgical planning in blepharoptosis, but traditional manual assessment using pen torch and ruler is subject to inter-observer variability and lack of standardisation. We evaluated an artificial intelligence-enabled virtual reality eye-tracking system to provide an objective alternative to current manual methods. In 101 patients with suspected blepharoptosis, the BulbiCAM device employed high-speed infrared video-oculography with convolutional neural network algorithms to automatically detect the pupil centre, corneal glint, and eyelid margins. When benchmarked against standard clinical assessment to establish validity, automated measurements showed high agreement, with mean differences of + 0.29 mm for upper eyelid position and + 0.01 mm for lower eyelid position. Critically, the device demonstrated high precision, with test-retest repeatability in 36 patients showing mean differences of − 0.03 mm and + 0.27 mm between visits. Successful acquisition was achieved in all participants with a median of one attempt, and 98% reported the device as comfortable and easy to use. This study demonstrates that AI-enabled VR eye-tracking matches the clinical accuracy of current standards while offering the repeatability and objectivity required to overcome the limitations of manual assessment.

**Supplementary Information:**

The online version contains supplementary material available at 10.1038/s41598-026-48931-3.

## Introduction

Blepharoptosis (ptosis) presents significant functional and cosmetic challenges for patients, often necessitating surgical intervention. Accurate assessment of eyelid position is therefore essential for diagnosis, classification, and surgical planning. To measure this eye-lid position two clinical metrics are used: Margin Reflex Distance 1 (MRD1), the vertical distance from the central corneal light reflex to the upper eyelid margin, and Margin Reflex Distance 2 (MRD2), the corresponding distance to the lower eyelid margin.

Traditionally, MRD1 and MRD2 are measured manually using a pen torch and a millimetre ruler. Although widely regarded as the clinical reference standard, this method is inherently subjective and prone to inter- and intra-observer variability^1^. Measurement accuracy may be influenced by examiner experience, ambient illumination, parallax error from ruler positioning, and patient factors such as inconsistent fixation or compensatory brow elevation. In routine clinical settings, limited standardisation of head position^2^ and lighting further complicates longitudinal comparison.

Digital imaging and computer-assisted methods have been introduced to overcome these limitations^3–5^, and VR-based approaches are especially promising^3,6^ as they can, control the visual environment, provide a fixed light source to generate the corneal reflex, utilise infrared tracking to monitor the pupil centre, and maintain a constant camera-to-eye distance.^7,8^ The BulbCam is one VR-based system, that uses convolutional neural network algorithms to detect the pupil centre, corneal glint and eyelid margins.^9^ By isolating the patient from external visual distractions, this platform may offer a more objective and reproducible eyelid measurements than conventional manual assessment. However, its performance has not previously been evaluated in patients with ptosis.

In this study, we assessed the agreement between BulbiCAM-derived MRD1 and MRD2 measurements and standard clinician-derived measurements in patients referred with suspected blepharoptosis. We also examined the repeatability of the automated system and explored its feasibility and acceptability in routine oculoplastics practice.

## Methods

### Study design and ethical approval

**Fig. 1:**
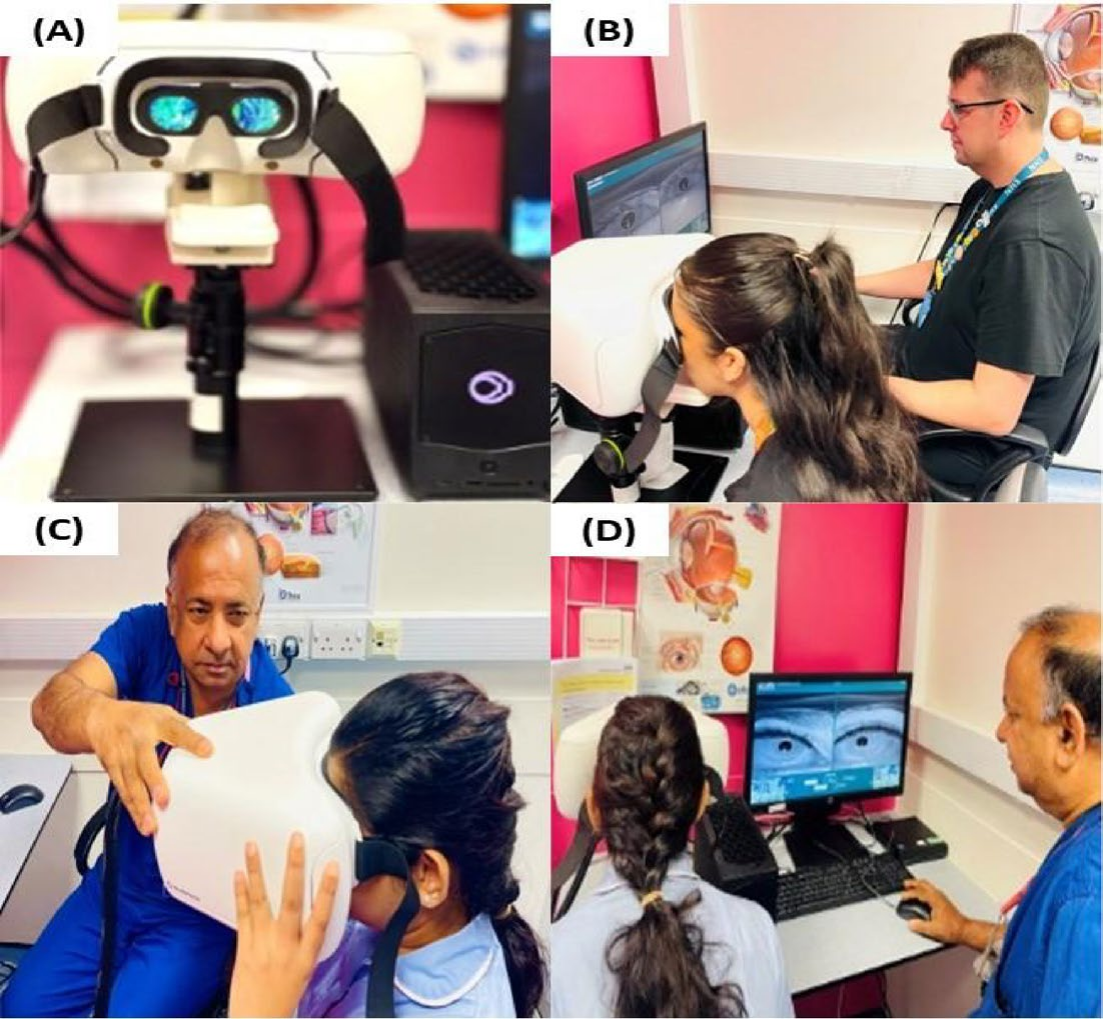
Fixed and handheld configurations of the BulbiCAM VR eye-tracking device. (**A**) The BulbiCAM headset mounted on a fixed desktop platform. (**B**,**D**) Standard clinical workflow used in this study, in which patients were assessed using the fixed desk-mounted configuration while the operator monitored the live acquisition interface. (**C**) Demonstration of an alternative handheld configuration, which was not used in the present study but may be useful for patients unable to access a desk or chin rest.

**Fig. 2:**
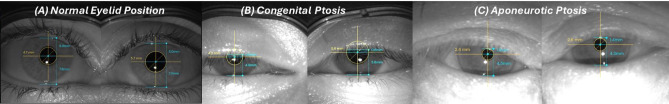
MRD1 and MRD2 measurements generated by the BulbiCAM VR eye-tracking system. Example infrared images illustrating eyelid margin and pupil segmentation across different clinical presentations. (**A**) Normal eyelid position, with detection of the corneal reflex, pupil centre, and upper and lower eyelid margins, producing typical MRD1 and MRD2 values. (**B**) Congenital ptosis, demonstrating markedly reduced MRD1 and an absent lid crease. (**C**) Aponeurotic ptosis, showing a characteristic moderate-to-severe reduction in MRD1 with a high lid crease. In all panels, the device’s convolutional-neural-network-based algorithms identify key landmarks (pupil centre, corneal glint, eyelid margins) and compute MRD1/MRD2 distances.

This prospective observational study was conducted in the Oculoplastic Unit at Leicester Royal Infirmary, University Hospitals of Leicester NHS Trust, United Kingdom. An overview of the study design is provided in Supplementary Fig. 1. The study adhered to the tenets of the Declaration of Helsinki and received approval from the NHS Research Ethics Committee (East Midlands - Leicester Central Research Ethics Committee, REC reference: 20/EM/0040, IRAS Project ID: 261121). Written informed consent was obtained from all participants prior to enrolment. Additional informed consent was obtained for the publication of images of participants and/or examiners included in Figs. 1 and 2. Data collection was undertaken between 1st July 2025 and 26th September 2025.

### Participants

Consecutive patients referred to the Oculoplastic Unit with suspected blepharoptosis were invited to participate. Eligible individuals were required to be aged 5 years or older (based on prior work with age limitations using VR headsets)^10^ and able to maintain primary gaze during image acquisition with the VR headset. Exclusion criteria included inability to wear the device comfortably, active ocular surface disease that could interfere with measurements. A subset of 36 participants, encompassing a broad range of ptosis aetiologies, was invited for a second visit (> 2 weeks after the initial assessment) to evaluate the test-retest repeatability of VR measurements.

### Clinical measurements (standard of care)

Clinical MRD1 and MRD2 measurements were performed by multiple experienced clinicians within the oculoplastic unit, including four consultant-grade and three senior fellow/senior registrar-grade oculoplastic surgeons, using a standardised ruler-based method. Brow position was not objectively controlled using mechanical fixation; rather, examiners visually monitored and minimised frontalis overaction in accordance with standard clinical practice. Patients were seated with the head in primary position and instructed to fixate on a distant target to minimise accommodation and brow recruitment. A handheld pen torch was used to generate the central corneal light reflex, and a millimetre ruler was positioned in the vertical plane immediately adjacent to the eyelid margin. MRD1 was defined as the distance from the corneal light reflex to the midpoint of the upper eyelid margin, and MRD2 as the corresponding distance to the lower eyelid margin. Lighting conditions were kept consistent, and examiners were instructed to monitor for compensatory frontalis activation. Measurements were taken for each eye and represented the clinical reference standard for subsequent agreement analyses.

### VR (BulbiCAM) measurement protocol

Following clinical assessment, participants underwent automated eyelid measurements using the BulbiCAM device (Bulbitech AS, Trondheim, Norway), a non-invasive virtual reality (VR) head-mounted system (Fig. 1). The device can be operated in either a fixed or handheld configuration; for this study, only the fixed configuration was used to ensure maximal stability and standardisation across participants.

The system incorporates an infrared (IR) video-oculography module with a high-speed camera operating at 400 frames per second, alternating between bright-pupil and dark-pupil illumination. Custom lens holders angled 10° vertically and 7° horizontally minimise IR glare and optimise alignment with the visual axis. A silicone face mask was used to block ambient light and maintain consistent head positioning.

During acquisition, a 3D visual target (a garden scene) was displayed at a virtual distance corresponding to optical infinity to minimise accommodation and encourage stable fixation in primary gaze. The device’s embedded convolutional neural network (CNN) algorithms automatically detected the pupil centre, corneal glint (small white light points projected onto the corneal surface by an infrared light source), and eyelid margins. The device’s embedded convolutional neural network (CNN) automatically segments key ocular structures from the infrared video frames. The model is based on a U-Net architecture comprising four encoder blocks with feature channels increasing from 16 to 128, three decoder blocks with transposed convolutions, skip connections between corresponding encoder and decoder stages, and batch normalisation after each convolutional layer. The network outputs six semantic classes: background, glint, iris, pupil, sclera, and skin, from which the pupil centre, corneal glint, and upper and lower eyelid margins are identified.

The model was trained on 1,056 grayscale eye images acquired in eyelid-tracking mode, standardised to 400 × 1000 pixels and expanded to 2,108 effective training samples after two-fold oversampling. Data augmentation included random scaling (plus or minus 20%), rotation (plus or minus 35 degrees), flipping, colour and intensity perturbation, and multiplicative noise. Training used the Adam optimiser (learning rate 1 × 10^− 4^, batch size 12, maximum 2,000 epochs) with early stopping (patience 100 epochs). Ground-truth segmentation masks were generated by manual annotation using the Computer Vision Annotation Tool (CVAT), followed by visual quality review. The model was validated on a held-out cohort of 127 images acquired from a separate patient group, achieving a mean Intersection over Union (mIoU) of 0.856 across the six segmentation classes.

MRD1 and MRD2 were calculated from the vector displacement between the corneal glint and the respective eyelid landmarks. Frames affected by blinking, loss of fixation, or artefacts were automatically excluded by the system’s internal quality-control filters, ensuring that only high-quality frames contributed to the final measurement. Brow position was not objectively controlled using mechanical fixation during VR acquisition. However, the measurement algorithm calculates MRD1 and MRD2 from the corneal glint and eyelid margins and is therefore inherently independent of brow position.

### Feasibility and patient-reported outcome assessment

Feasibility of the VR-based measurement procedure was evaluated using both operator-recorded metrics and participant-reported experience measures.

### Operator-recorded feasibility

During each session, the examiner documented the number of attempts required to obtain a successful MRD measurement using the BulbiCAM device. An “attempt” was defined as a full acquisition cycle initiated by the operator; incomplete or aborted acquisitions were also counted. This metric was used to assess the practical ease, speed and reliability of obtaining automated measurements in a routine clinic setting.

### Participant-reported experience

Patient experience and perception of the technology was evaluated using a purposely designed questionnaire administered immediately post-assessment (Supplementary Table 1). The instrument was developed specifically for this study to capture domains of particular relevance to VR-based ophthalmic assessment, namely overall satisfaction, comfort, perceived ease and efficiency of the measurement process, and clarity of instructions, that are not addressed by existing generic usability scales. These domains were selected to reflect the key determinants of clinical acceptability for a novel point-of-care device whilst keeping the instrument brief enough for routine clinic use. Participants rated the device on a standard scale on the following four domains:

1. Overall satisfaction: to assess participants’ general impression of using the VR headset.
2. Comfort whilst wearing the headset.
3. Perceived ease and efficiency of the measurement process: capturing whether participants felt the acquisition process was quick and straightforward.
4. Clarity and ease of instructions for the ptosis measurement task: evaluating participants’ ability to understand and comply with the instructions displayed within the VR environment.

All questionnaires were completed immediately following the VR acquisition to minimise recall bias. Responses were collected anonymously and analysed descriptively.

### Statistical analysis

Statistical analyses were performed for right eyes, left eyes, and pooled (both eyes combined). Pooled estimates are reported as the primary analysis, as pooling increases measurement precision and is consistent with comparable ophthalmic method-comparison studies.^5,11^ Individual eye-level results are presented in the Supplementary Material (Supplementary Figs. 2 to 9) to verify that within-subject correlation has not materially influenced the pooled findings.

Agreement between clinician-derived and VR-derived MRD1 and MRD2 values was assessed using Bland-Altman analysis.^12^ Mean differences (bias) and 95% limits of agreement (LoA) were calculated. Since ptosis measurements were not normally distributed (Shapiro-Wilk test, *P* < 0.0001), Spearman’s rank correlation coefficients were calculated to assess the relationship between methods. Scatter plots were generated to visualise the correlation between clinical and VR-based measurements.

Sample size was determined a priori using data from Bodnar et al. (2016), who reported an SD of differences of 0.347 mm for MRD1 and 0.342 mm for MRD2. Using a maximum allowable difference of 1.0 mm (based on clinical grading of ptosis severity in 1 mm increments), a significance level of 0.05, and a 95% agreement level, the minimum required sample size at 90% power was 50 participants for MRD1 and 93 for MRD2 (calculated using the blandPower package in R 4.5.1). Our study of 101 participants (202 eyes) exceeds these estimates.

For participants who underwent a repeat VR assessment, test-retest repeatability was evaluated using Bland-Altman plots comparing Visit 1 and Visit 2 measurements. Mean differences and 95% LoA were calculated to quantify repeatability. Intraclass correlation coefficients (ICC, two-way random, single measures, absolute agreement) were computed for each eye separately.

Feasibility outcomes, including the number of acquisition attempts and participant questionnaire responses, were summarised descriptively. All statistical analyses were performed using JASP 0.19.3.

## Results

### Demographics and clinical characteristics

A total of 101 participants (202 eyes) were included in the study (Table 1). The mean age was 59.7 ± 18.0 years (range: 13–89 years). Of these, 59 participants (58.4%) were female and 42 (41.6%) were male. Participants presented with a spectrum of ptosis subtypes (Table 1). Exemplar BulbiCAM imaging across ptosis subtypes is shown in Fig. 2. A predefined subgroup of participants (*n* = 36) returned for a second session to undergo repeat VR measurements for the repeatability analysis.

**Table 1:** Participant demographics and clinical characteristics (*N* = 101).

Characteristic	*n* or mean ± SD	% or range
Total participants	101	–
Total eyes	202	–
Age (years)	59.7 ± 18.0	13–89
Sex		
Female	59	58.4%
Male	42	41.6%
Ptosis subtype		
Aponeurotic	54	53.5%
Mechanical	19	18.8%
Pseudoptosis	14	13.9%
Myogenic/Neurogenic	11	10.9%
Congenital	2	2.0%
Traumatic	1	1.0%
Repeatability subgroup (n)	36	–

SD, standard deviation. Values are presented as n (%) for categorical variables and mean ± SD (range) for continuous variables.

### Agreement between clinical and VR measurements

**Fig. 3:**
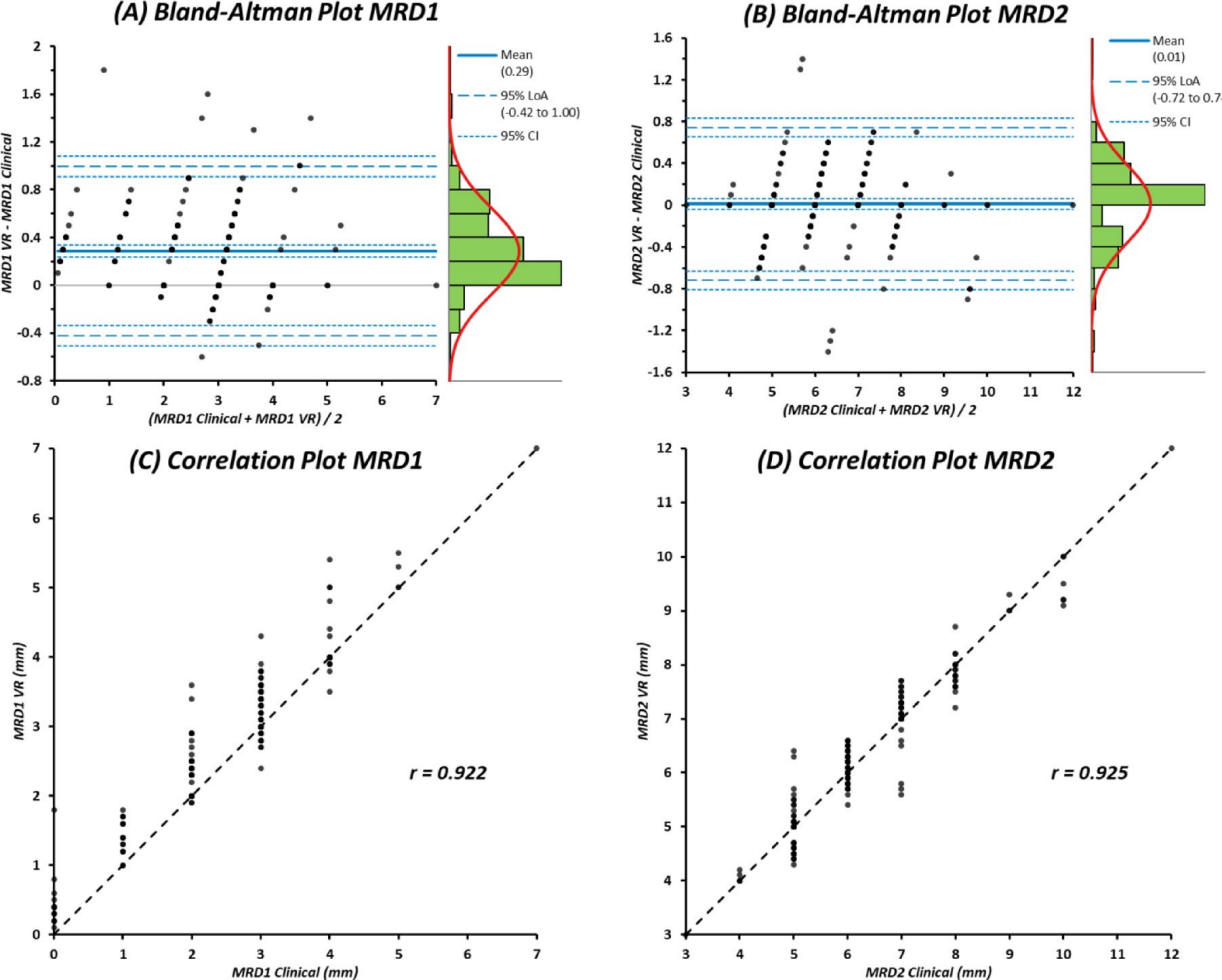
Agreement between clinical measurements and VR-based automated measurements for MRD1 and MRD2. (**A**) Bland-Altman plot showing agreement between clinical and VR-derived MRD1 measurements (combined eyes). The mean bias and 95% limits of agreement are displayed with corresponding distribution histograms. (**B**) Bland-Altman plot for MRD2 demonstrating similarly minimal systematic bias and acceptable limits of agreement. (**C**) Correlation analysis for MRD1 showing strong linear association between the clinical and VR measurements. (**D**) Correlation analysis showing strong correlation between clinical and VR derived MRD1/2 measurements.

**Fig. 4:**
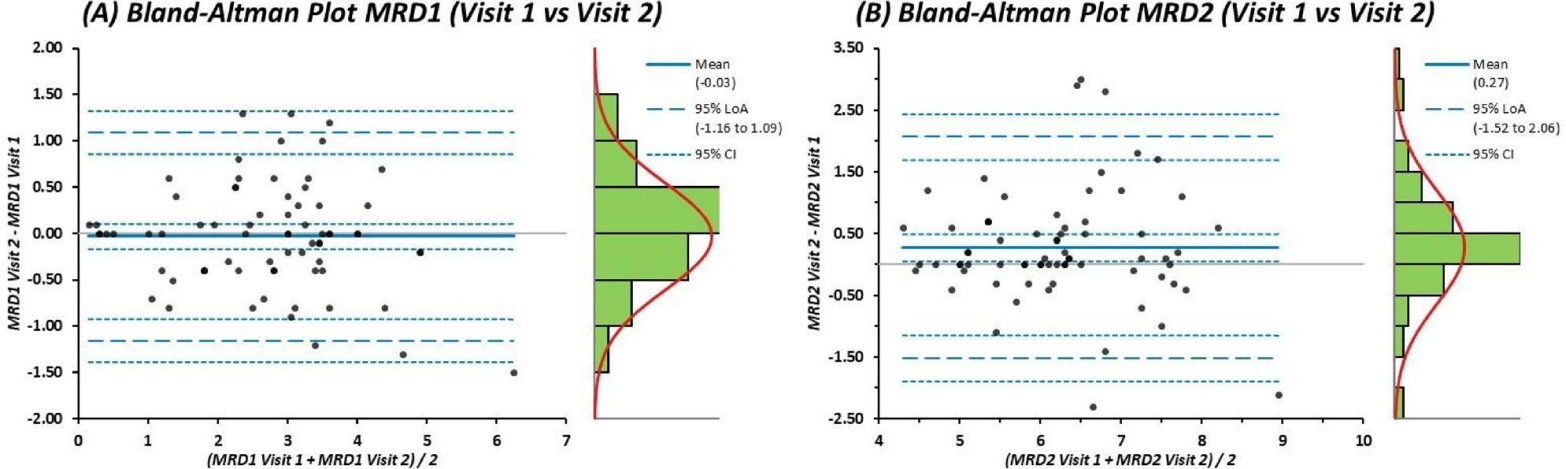
Test–retest repeatability of BulbiCAM measurements (Visit 1 vs. Visit 2). (**A**) Bland-Altman plot showing the difference in MRD1 measurements between Visit 1 and Visit 2 plotted against their mean. The solid line represents the mean difference (− 0.03 mm), with dashed lines indicating the 95% limits of agreement (-1.16 to + 1.09 mm). (**B**) Bland-Altman plot showing the difference in MRD2 measurements between Visit 1 and Visit 2. The mean difference was + 0.27 mm, with 95% limits of agreement ranging from − 1.52 to + 2.06 mm. For both panels, right-hand density histograms display the distribution of measurement differences with overlaid 95% confidence intervals. These plots demonstrate good overall repeatability of automated VR-derived eyelid measurements despite expected biological and behavioural variability.

Agreement between clinician-derived and VR-derived MRD measurements is summarised in Table 2, with Bland-Altman plots and scatter plots shown in Fig. 3 (pooled eyes) and Supplementary Figs. 2 to 5 (individual eyes). For MRD1, the mean difference between methods was + 0.29 mm, indicating that VR measurements were slightly higher on average than clinical values. The 95% limits of agreement ranged from − 0.42 to + 1.00 mm, with no evidence of proportional bias on visual inspection of the Bland-Altman plot (Fig. 4A). Strong correlation was observed between clinical and VR derived MRD1 measurements (*r* = 0.922; *P* < 0.0001; Fig. 4C). For MRD2, the mean difference was + 0.01 mm, with 95% limits of agreement from − 0.72 to + 0.74 mm (Fig. 4B). As with MRD1, no systematic proportional bias was observed. Correlation between clinical and VR MRD2 measurements was similarly high (*r* = 0.925; *P* < 0.0001; Fig. 4D).

**Table 2:** Summary of agreement statistics for MRD1 and MRD2.

Analysis	Measure	Mean bias (mm)	95% LoA (mm)
Agreement: clinical vs. VR (*n* = 101 participants, 202 eyes)			
Pooled eyes	MRD1	+ 0.29	−0.42 to + 1.00
	MRD2	+ 0.01	−0.72 to + 0.74
Right eye	MRD1	+ 0.30	−0.38 to + 0.98
	MRD2	+ 0.03	−0.74 to + 0.80
Left eye	MRD1	+ 0.27	−0.47 to + 1.01
	MRD2	+ 0.00	−0.69 to + 0.69

LoA, limits of agreement; MRD1, margin reflex distance 1; MRD2, margin reflex distance 2; VR, virtual reality.

### Repeatability of VR measurements

Repeatability of the VR-derived measurements was assessed in participants who underwent a second acquisition session (Table 3; Fig. 4 and Supplementary Figs. 6 to 9). The VR device demonstrated good test-retest repeatability. MRD1 showed a mean difference of −0.03 mm (LoA: −1.16 to + 1.09 mm), and MRD2 a mean difference of + 0.27 mm (LoA: −1.52 to + 2.06 mm). No proportional bias was observed. ICC analysis demonstrated excellent test-retest reliability for MRD1 (right eye: 0.915, 95% CI: 0.833 to 0.958; left eye: 0.903, 95% CI: 0.810 to 0.952). MRD2 ICCs were lower (right eye: 0.641, 95% CI: 0.379 to 0.808; left eye: 0.825, 95% CI: 0.670 to 0.911), consistent with the restricted between-subject variability in lower eyelid position in this predominantly ptosis cohort (Supplementary Table 2).

**Table 3:** Summary of repeatability statistics for MRD1 and MRD2.

Analysis	Measure	Mean Bias (mm)	95% LoA (mm)
Repeatability: VR Visit 1 vs. Visit 2 (*n* = 36 participants, 72 eyes)			
Pooled eyes	MRD1	−0.03	−1.16 to + 1.09
	MRD2	+ 0.27	−1.52 to + 2.06
Right eye	MRD1	−0.03	−1.13 to + 1.08
	MRD2	+ 0.11	−2.52 to + 2.74
Left eye	MRD1	−0.04	−1.20 to + 1.11
	MRD2	+ 0.25	−1.79 to + 2.28

LoA, limits of agreement; MRD1, margin reflex distance 1; MRD2, margin reflex distance 2; VR, virtual reality.

### Feasibility and patient reported outcomes

Feasibility of the VR-based acquisition was high. A successful MRD measurement was obtained in all participants. The median number of attempts required to achieve a valid acquisition was 1 (range: 1–4).

Participant-reported experience with the device was favourable. Most respondents rated their overall experience with the VR headset as “Very Good” or “Good” (*n* = 99, 98%). The majority of participants reported that the headset was comfortable to wear (*n* = 99, 98%), and most felt that the acquisition process was quick and straightforward (*n* = 94, 93%). Similarly, most participants indicated that the instructions for completing the ptosis measurement task were easy to follow (*n* = 99, 98%). No adverse events or intolerance to the VR headset were reported.

## Discussion

In this prospective method-comparison study, we demonstrated that an AI-enabled virtual-reality eye-tracking device can provide automated measurements of MRD1 and MRD2 with high agreement compared with standard clinician-derived assessments. VR-derived measurements showed minimal mean bias for both MRD1 (+ 0.29 mm) and MRD2 (+ 0.01 mm), with narrow limits of agreement and strong linear correlations. Importantly, the system also exhibited good repeatability, with mean differences close to zero across repeated sessions. Feasibility was high, with successful acquisitions in all participants, a median of one attempt required, and favourable patient-reported comfort and usability.

Previous studies have developed comparable deep learning-based systems to measure MRD1 and MRD2 measurements systematically. Lou et al. demonstrated excellent correlation between automated image-based MRD measurements and manual measurements in 135 eyes with blepharoptosis, reporting a bias range of 0.09 to 0.15 mm for MRD1.^3^ Similarly, Van Brummen and colleagues developed PeriorbitAI, an AI segmentation algorithm achieving mean absolute differences of 0.5 mm for MRD1 and MRD2 when compared with human graders.^11^ More recently, Rana et al. reported mean absolute errors ranging from 0.22 mm to 0.88 mm for various periocular measurements using a deep learning facial landmark detection network, with excellent intraclass correlation coefficients (ICC) (> 0.90) for MRD1 and MRD2^13^. Similarly, Ahemiti et al. also demonstrated a small bias range from 0.003 to 0.14 mm and excellent ICC of 0.932 using deep learning-based adaptive and automatic measurement (DeepAAM) as compared with manual measurements of MRD1 and MRD2.^6^ Our results strongly align with these findings, demonstrating mean biases of + 0.29 mm (MRD1) and + 0.01 mm (MRD2).

We also demonstrated narrow 95% limits of agreement (-0.42 to + 1.00 mm for MRD1; -0.72 to + 0.74 mm for MRD2) in our study. These findings are in line with. Cao et al. who reported slightly larger mean absolute differences for MRD1 (approximately 0.5 mm) using a deep learning network applied to 2D photographs^14^, whilst Bodnar et al. reported mean difference of 0.03 mm for and 0.13 mm for MRD1 and MRD2 respectively.^5^ Taken together, this confirms that the BulbiCAM offers sufficient precision for clinical decision-making, as the sub-millimetre variations observed are negligible in the context of surgical planning, which typically operates at a millimetre scale. Nevertheless, these limits warrant careful consideration in clinical scenarios involving borderline surgical decision-making, where MRD1 differences of less than 1 mm may influence the decision to operate or the choice of surgical technique. Similarly, when monitoring postoperative outcomes, changes within the limits of agreement should be interpreted with caution, as they may reflect measurement variability rather than genuine anatomical change.

The test-retest repeatability of the BulbiCAM system was similarly good, with mean differences of -0.03 mm for MRD1 and + 0.27 mm for MRD2 between visits. MRD1 demonstrated excellent intraclass correlation coefficients (ICC > 0.90 bilaterally), confirming strong relative reliability for upper eyelid measurements. MRD2 ICCs were lower (0.641 to 0.825); however, ICC is a measure of relative reliability that is sensitive to the spread of values across the sample. Because ptosis predominantly affects the upper eyelid, MRD2 values in our cohort were relatively homogeneous, and when between-subject variance is restricted, ICC is deflated irrespective of device precision. Conditions associated with greater MRD2 variability, such as thyroid eye disease with lower lid retraction, were represented by very few participants in the repeatability subgroup. The Bland-Altman limits of agreement, which quantify absolute measurement precision independently of sample composition, therefore provide the more clinically informative assessment of device repeatability in this context. These findings demonstrate that the automated system can provide consistent and reproducible measurements across sessions, an important consideration for longitudinal monitoring of ptosis progression or postoperative outcomes. Furthermore, BulbiCAM’s performance matches the clinical reliability established by Schulz et al., who reported comparable precision (95% limits of agreement of ± 1.1 mm) for automated MRD1 measurements in oculofacial disorders.^15^.

The VR-based approach offers several potential advantages over both traditional manual measurement and 2D photographic analysis. First, the controlled visual environment eliminates ambient lighting variability and provides a standardised fixation target at optical infinity, minimising accommodation and promoting stable primary gaze. Traditional photography-based methods depend on consistent camera positioning, distance, and lighting conditions, which can be difficult to standardise across sessions and institutions. By contrast, the VR headset provides a rapid, standardised, point-of-care solution that functions as an out-of-the-box measurement system requiring no additional calibration or specialist imaging equipment. Image acquisition and MRD calculation occur within a single integrated step, eliminating the need for offline image processing, pixel-to-millimetre calibration, and multi-step analytical workflows that are typically required for photographic methods.

Second, the infrared illumination used by the BulbiCAM system as opposed to visible light from handheld pen torches, reduces reflex blinking, photophobia and provides consistent generation of the corneal glint. Third, the fixed camera-to-eye distance inherent in the head-mounted design eliminates magnification variability and parallax error. Finally, the automated ocular landmark detection using convolutional neural networks removes inter-observer variability, with prior studies demonstrating excellent reliability for AI-based periocular measurements.^11,13^.

The high agreement and repeatability of VR-derived measurements suggest potential applications in both clinical practice and research. For surgical planning, objective and reproducible measurements could improve preoperative assessment and facilitate prediction of surgical outcomes. Digital image analysis has been shown to provide novel metrics for capturing functional and aesthetic outcomes after eyelid surgery, enhancing the analysis of postoperative results. The rapid acquisition time (median of one attempt) and favourable patient experience support the feasibility of incorporating this technology into routine clinical workflow. For research applications, standardised automated measurement could reduce sample size requirements in clinical trials by minimising measurement error. The ability to perform remote or self-administered assessments using portable VR headsets could facilitate telemedicine applications and home monitoring of ptosis progression. Automated periocular measurements may help increase the objectivity of clinical assessments and facilitate remote evaluation of patients through telehealth platforms.

Recent developments in automated periocular measurement include both semi-automated tools requiring user input and fully automated systems. Peterson et al. compared a semi-automated FIJI-based macro (OrbitJ)^16^ with two fully automated deep learning algorithms, reporting excellent agreement (ICC > 0.8) between OrbitJ and manual measurements, but with analysis times of 5.4 min per image compared to 1.45 s for their automated OrbitMap algorithm. Our VR-based approach achieves real-time automated measurement during a brief acquisition session, combining the accuracy of manual measurement with the efficiency of fully automated analysis.

Several limitations should be acknowledged. First, our study compared VR measurements against manual clinical assessment as the reference standard. However, manual measurement itself has inherent variability and may not represent an absolute ground truth. Clinical measurements were performed by multiple oculoplastic clinicians (four consultants and three fellows/registrars) and a formal inter-observer reliability analysis was not undertaken, as the primary aim was to compare the VR method against standard clinical assessment as practised in routine care. Some degree of inter-examiner variability is expected with manual ruler-based measurement, but the involvement of multiple clinicians at different grades more closely reflects the real-world clinical setting in which this technology would be deployed. Brow position was not objectively controlled using mechanical fixation during either clinical or VR assessment. While examiners monitored and minimised frontalis overaction in accordance with standard practice, and the VR measurement algorithm itself is independent of brow position as it derives MRD from the corneal glint and eyelid margins, involuntary frontalis recruitment may still secondarily elevate the upper eyelid, introducing a potential source of variability in both measurement modalities. Furthermore, the current system measures MRD1 and MRD2 in primary gaze only and does not assess levator function, which requires measurement of upper eyelid excursion across gaze positions and is essential for surgical planning. MRD1 and MRD2 nonetheless remain valuable as objective metrics for grading ptosis severity and monitoring change over time. Secondly, the study was conducted at a single centre with a specific patient population referred for oculoplastic evaluation. The generalisability to other populations, including paediatric patients, individuals with severe eyelid abnormalities, or those unable to cooperate with VR headset placement, requires further investigation. Although our inclusion criteria specified age ≥ 5 years, the youngest participant in this study was 13 years old.

Thirdly, while the VR system demonstrated high agreement for group-level measurements, individual-level agreement showed wider limits that may limit its use for detecting small changes in ptosis severity in individual patients. The 95% limits of agreement for MRD1 (-0.42 to + 1.00 mm) suggest that changes < 1 mm should be interpreted with caution. Furthermore, this study evaluated only the fixed desk-mounted configuration of the BulbiCAM device. The handheld configuration was not assessed but may offer different performance characteristics. The ergonomic advantage of the detachable, portable VR headset could extend applicability to patients unable to position themselves at a desk i.e., wheelchair bound patients or those with limited cervical mobility. Finally, the CNN segmentation model embedded in the BulbiCAM device was validated on a held-out internal cohort but has not undergone formal external validation on an independent dataset. While the segmentation performance (mIoU 0.856) and the clinical agreement reported in this study are encouraging, external validation across diverse populations and imaging conditions would further strengthen confidence in the generalisability of the automated measurements.

Future research could explore the integration of VR-based measurement with other AI applications in ophthalmology, including automated diagnosis of ptosis subtypes and prediction of surgical outcomes. The ability to capture dynamic eyelid position throughout the cardiac cycle or during various functional tasks (e.g., downgaze, blinking) could provide additional clinically relevant information beyond static measurements^15^ such as levator function testing. Development of normative databases stratified by age, sex, and ethnicity could enhance interpretation of VR-derived measurements and facilitate identification of pathological deviations. Integration with electronic health record systems could enable longitudinal tracking of ptosis progression and automated detection of clinically significant changes requiring intervention. Finally, prospective validation studies comparing VR measurements with postoperative outcomes would help establish the clinical utility of automated assessment in surgical planning and predicting the degree of correction required for optimal functional and aesthetic results.

## Conclusion

This study demonstrates that AI-enabled VR eye-tracking provides MRD1 and MRD2 measurements with high agreement to standard clinical assessment, good test-retest repeatability, and excellent feasibility in routine oculoplastics practice. The technology offers advantages over manual measurement including objective ocular landmark detection, elimination of inter-observer variability, and standardised acquisition conditions. These findings support the potential role of VR-based automated measurement in both clinical care and research applications for ptosis assessment, with particular promise for enhancing objectivity, efficiency, and accessibility of eyelid position evaluation.

## Supplementary Information

Below is the link to the electronic supplementary material.

Supplementary Material 1

## Data Availability

All data generated or analysed during this study are included in this published article (and its Supplementary Information files).
