# Supplementary material for: Artificial intelligence-driven virtual reality eye-tracking for the objective measurement of MRD1 and MRD2 in blepharoptosis
Source: Sci Rep. 2026 Apr 14;16:17430. doi: 10.1038/s41598-026-48931-3 (PMC13237289; doi:10.1038/s41598-026-48931-3)
Supplement: Supplementary file 1 — Supplementary Material 1 [file 41598_2026_48931_MOESM1_ESM.docx]

***Artificial Intelligence-Driven Virtual Reality Eye-Tracking for the Objective Measurement of MRD1 and MRD2 in Blepharoptosis***

Supplementary Material

***Table 1: BulbiCAM Device Participant-Reported Experience Questionnaire***

Based on your perception now, after undergoing the Virtual-reality Headset Ptosis Assessment, please answer the following questions of your experience by ticking the appropriate box.

|  | ***Very Good*** | ***Good*** | ***Poor*** |
| --- | --- | --- | --- |
| 1. *How was the overall device experience?* |  |  |  |
|  | ***Yes*** | ***No*** | ***Not Applicable*** |
| 1. *Were you comfortable whilst wearing the headset?* |  |  |  |
| 1. *Was the measurement process easy and efficient?* |  |  |  |
| 1. *Were the instructions for the ptosis measurement task clear and easy to follow?* |  |  |  |

***Table 2 Intraclass correlation coefficients for test-retest repeatability of VR-derived MRD1 and MRD2 measurements (n = 31 participants).***

| **Measure** | **ICC** | **95% CI** | **Interpretation** |
| --- | --- | --- | --- |
| **MRD1 right eye** | 0.915 | 0.833 to 0.958 | Excellent |
| **MRD1 left eye** | 0.903 | 0.810 to 0.952 | Excellent |
| **MRD2 right eye** | 0.641 | 0.379 to 0.808 | Moderate |
| **MRD2 left eye** | 0.825 | 0.670 to 0.911 | Good |


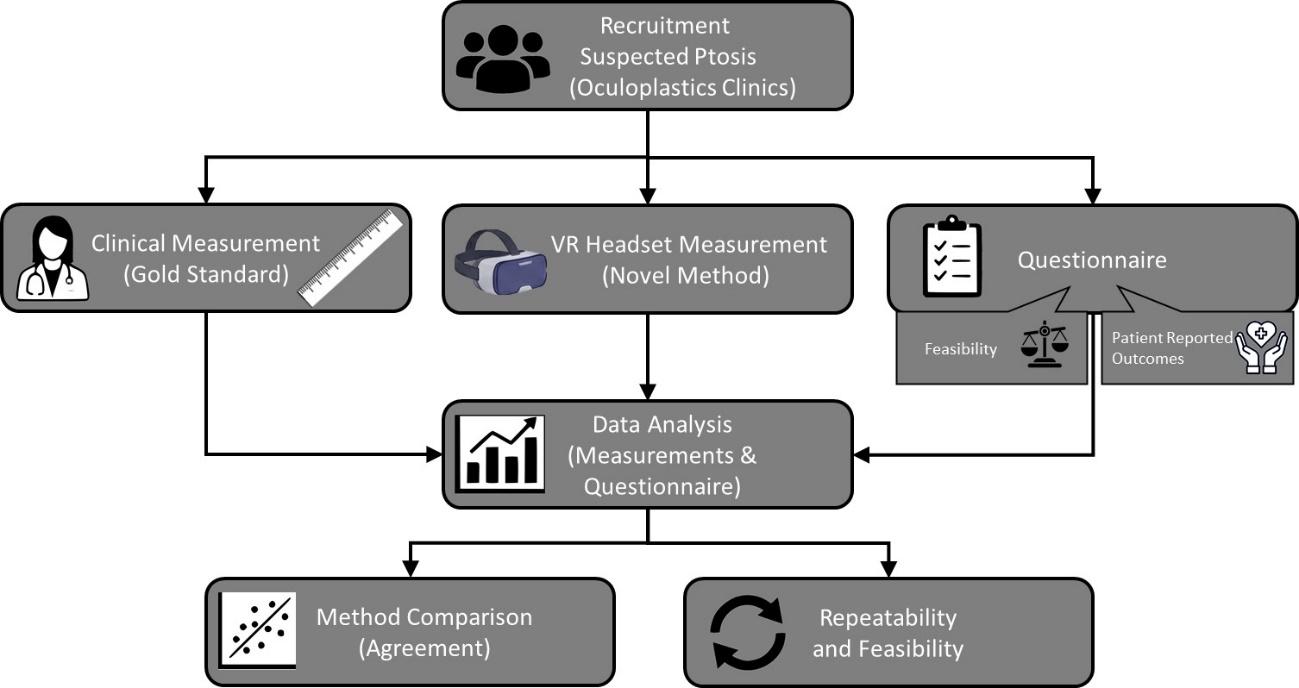


***Figure 1:*** *Study design overview. Flow diagram illustrating the prospective evaluation of an AI-driven VR eye-tracking system for ptosis assessment. Patients with suspected blepharoptosis were recruited from oculoplastics clinics and underwent (i) standard clinical measurements of eyelid position (gold standard), (ii) automated VR headset–based measurements (novel method), and (iii) a feasibility and patient-reported experience questionnaire. Outcomes included method comparison (agreement between VR and clinical measurements) and assessment of repeatability and feasibility of the VR system.*

***Figure 2: Bland-Altman and Scatter Plots MRD1 Right Eye (RE) (Clinical (C) Vs BulbiCAM (B))***


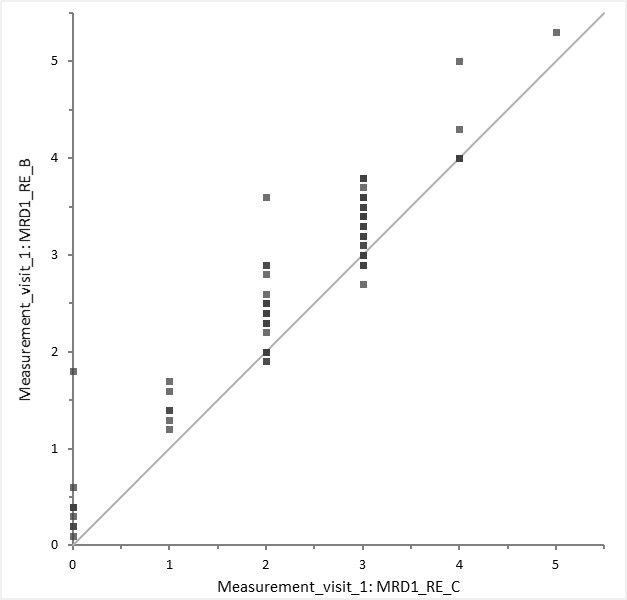


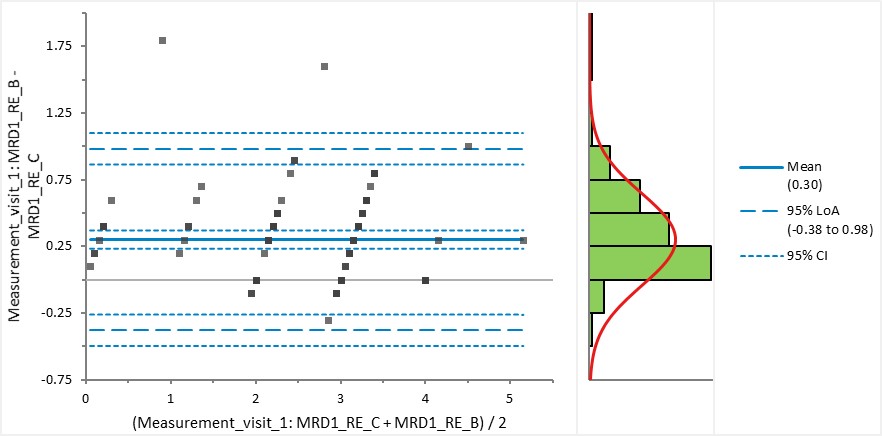


| Parameter | Estimate | 95% CI | | SE |
| --- | --- | --- | --- | --- |
| Mean difference | 0.30 | 0.235 | to 0.371 | 0.035 |
| 95% Lower LoA | -0.38 | -0.494 | to -0.259 | 0.059 |
| 95% Upper LoA | 0.98 | 0.865 | to 1.100 | 0.059 |

***Figure 3: Bland-Altman and Scatter Plots MRD1 Left Eye (LE) (Clinical (C) Vs BulbiCAM (B))***


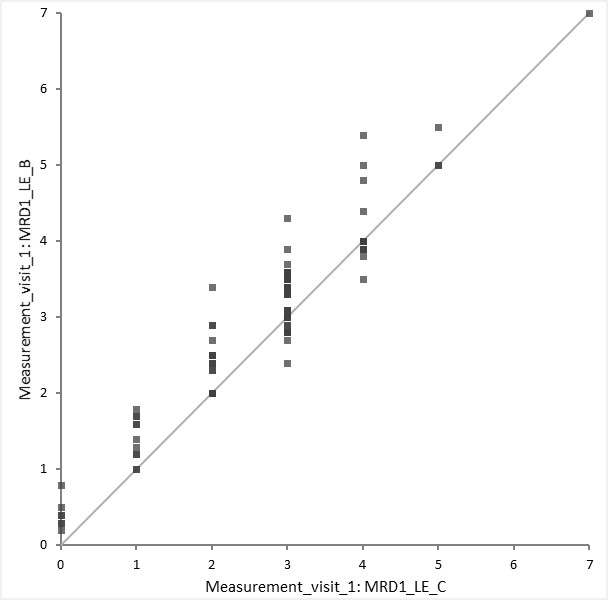


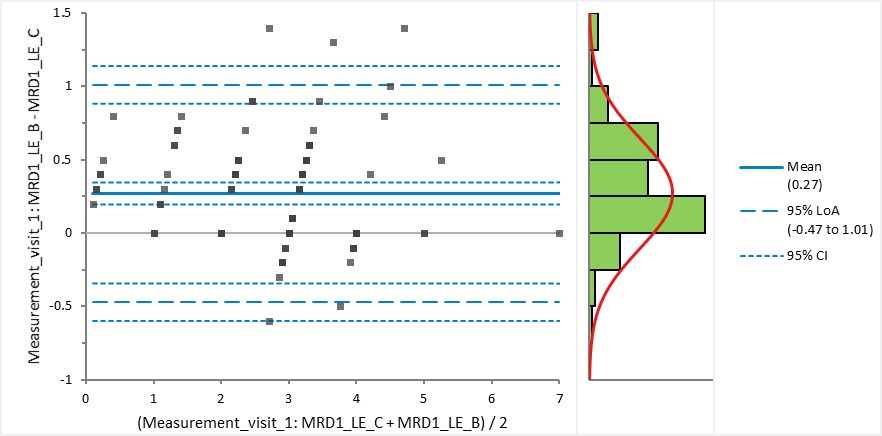


| Parameter | Estimate | 95% CI | | SE |
| --- | --- | --- | --- | --- |
| Mean difference | 0.27 | 0.197 | to 0.346 | 0.038 |
| 95% Lower LoA | -0.47 | -0.596 | to -0.340 | 0.064 |
| 95% Upper LoA | 1.01 | 0.883 | to 1.138 | 0.064 |

***Figure 4: Bland-Altman and Scatter Plots MRD2 Right Eye (RE) (Clinical (C) Vs BulbiCAM (B))***


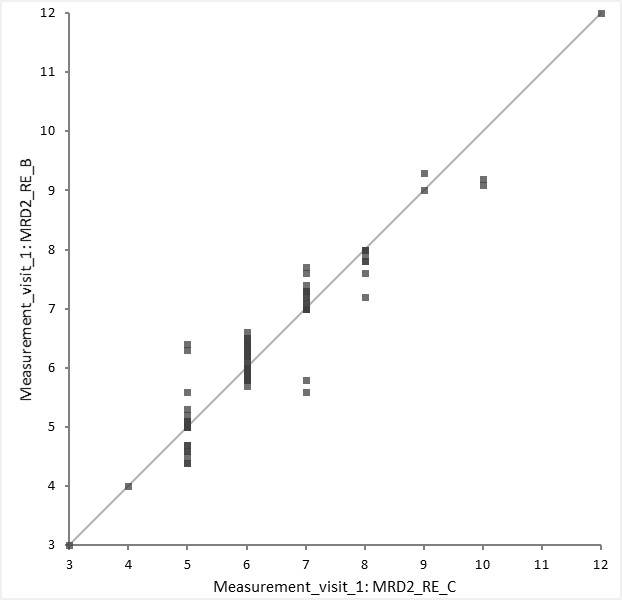


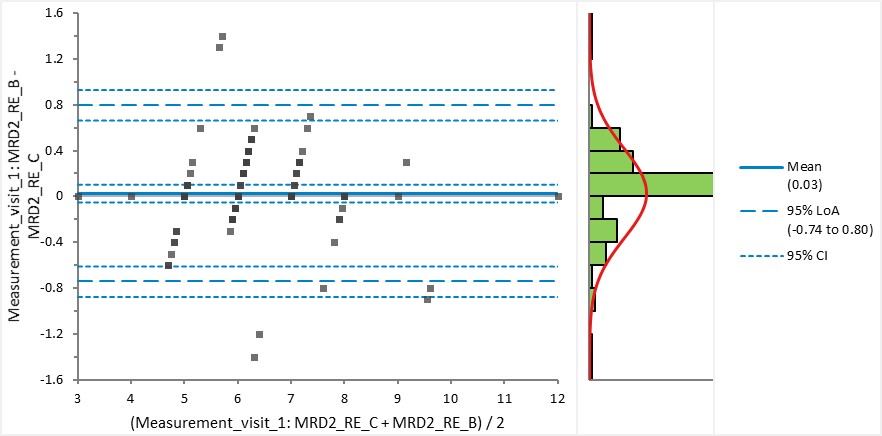


| Parameter | Estimate | 95% CI | | SE |
| --- | --- | --- | --- | --- |
| Mean difference | 0.03 | -0.050 | to 0.105 | 0.039 |
| 95% Lower LoA | -0.74 | -0.874 | to -0.609 | 0.067 |
| 95% Upper LoA | 0.80 | 0.664 | to 0.930 | 0.067 |

***Figure 5: Bland-Altman and Scatter Plots MRD2 Left Eye (LE) (Clinical (C) Vs BulbiCAM (B))***


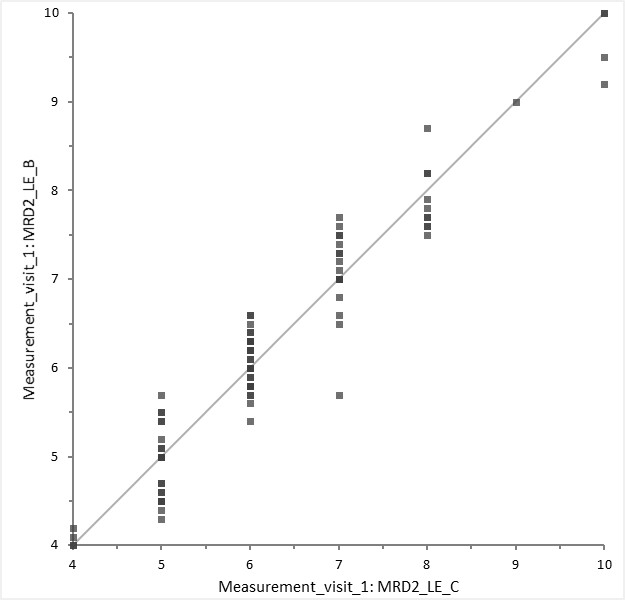


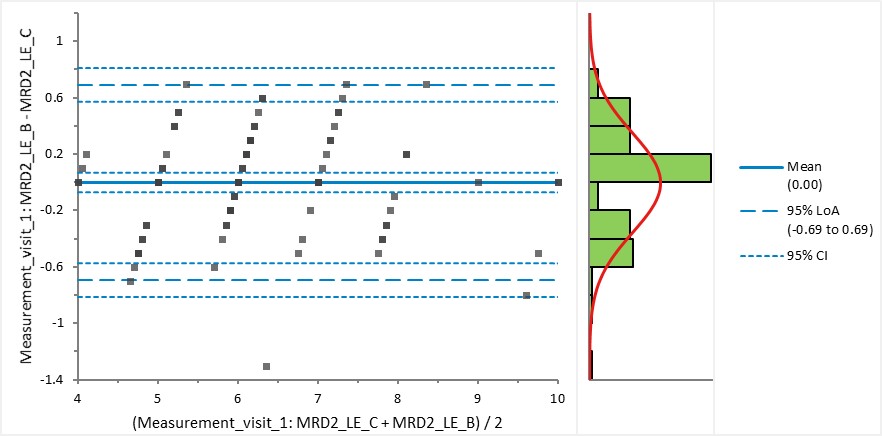


| Parameter | Estimate | 95% CI | | SE |
| --- | --- | --- | --- | --- |
| Mean difference | 0.00 | -0.072 | to 0.068 | 0.035 |
| 95% Lower LoA | -0.69 | -0.813 | to -0.574 | 0.060 |
| 95% Upper LoA | 0.69 | 0.570 | to 0.809 | 0.060 |

***Figure 6: Bland-Altman and Scatter Plots MRD1 Right Eye (RE) (Visit 1 Vs Visit 2)***


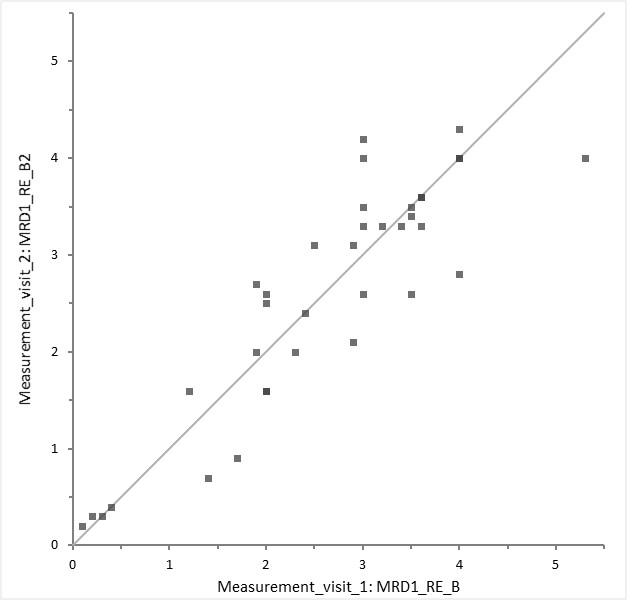


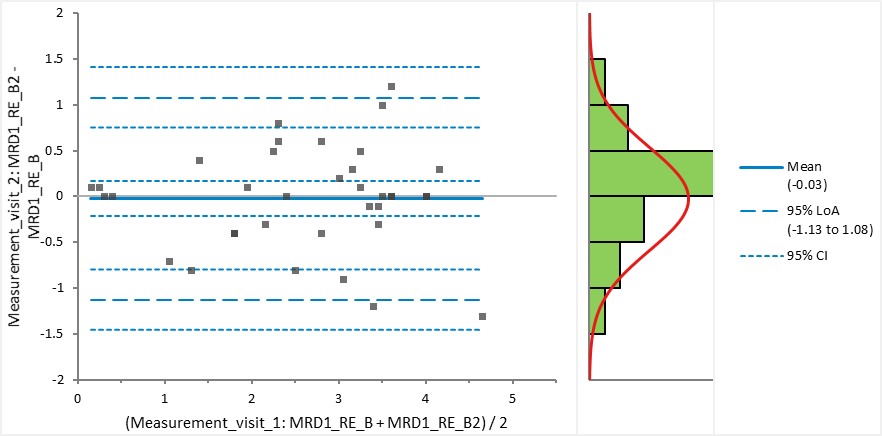


| Parameter | Estimate | 95% CI | | SE |
| --- | --- | --- | --- | --- |
| Mean difference | -0.03 | -0.215 | to 0.165 | 0.094 |
| 95% Lower LoA | -1.13 | -1.457 | to -0.800 | 0.162 |
| 95% Upper LoA | 1.08 | 0.750 | to 1.407 | 0.162 |

***Figure 7: Bland-Altman and Scatter Plots MRD1 Left Eye (LE) (Visit 1 Vs Visit 2)***

***
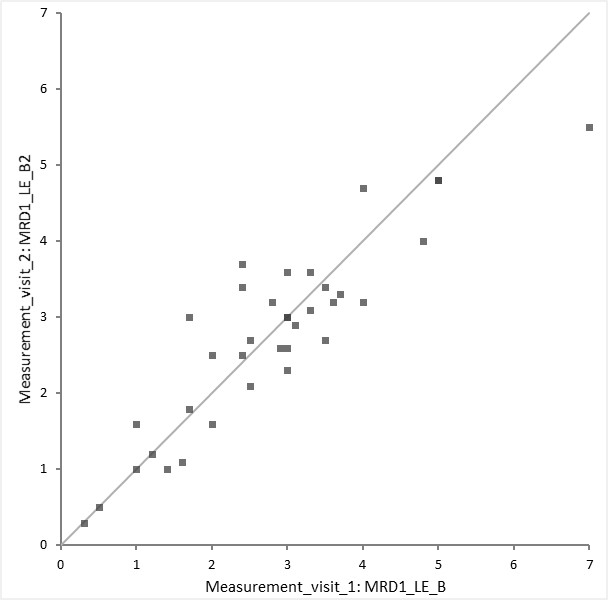
***

***
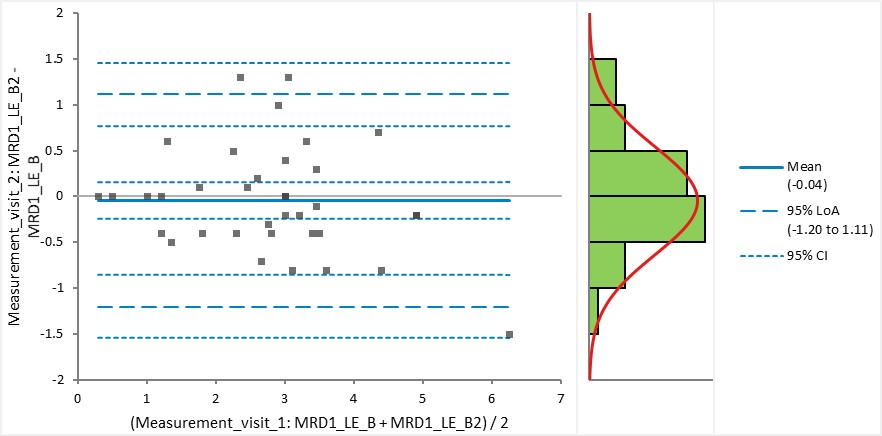
***

| Parameter | Estimate | 95% CI | | SE |
| --- | --- | --- | --- | --- |
| Mean difference | -0.04 | -0.244 | to 0.155 | 0.098 |
| 95% Lower LoA | -1.20 | -1.547 | to -0.857 | 0.170 |
| 95% Upper LoA | 1.11 | 0.768 | to 1.458 | 0.170 |

***Figure 8: Bland-Altman and Scatter Plots MRD2 Right Eye (RE) (Visit 1 Vs Visit 2)***


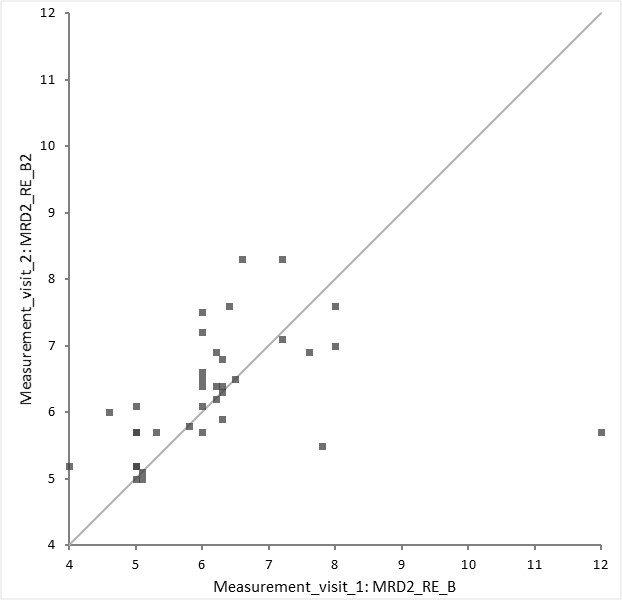


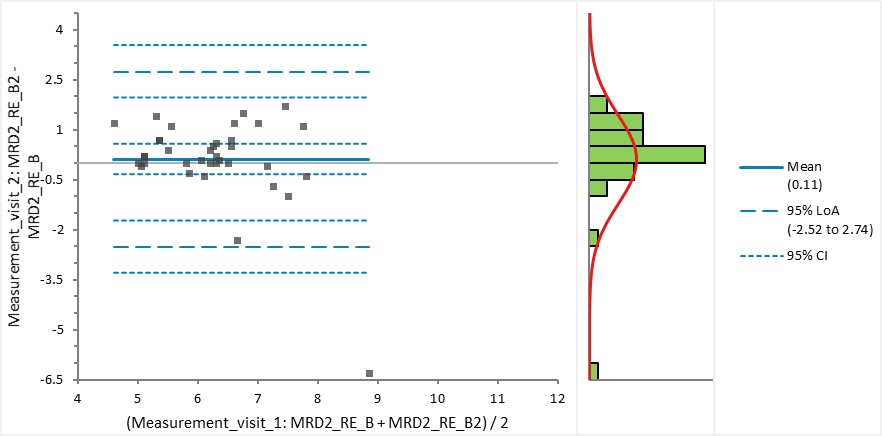


| Parameter | Estimate | 95% CI | | SE |
| --- | --- | --- | --- | --- |
| Mean difference | 0.11 | -0.340 | to 0.568 | 0.224 |
| 95% Lower LoA | -2.52 | -3.300 | to -1.734 | 0.386 |
| 95% Upper LoA | 2.74 | 1.961 | to 3.528 | 0.386 |

***Figure 9: Bland-Altman and Scatter Plots MRD2 Left Eye (LE) (Visit 1 Vs Visit 2)***


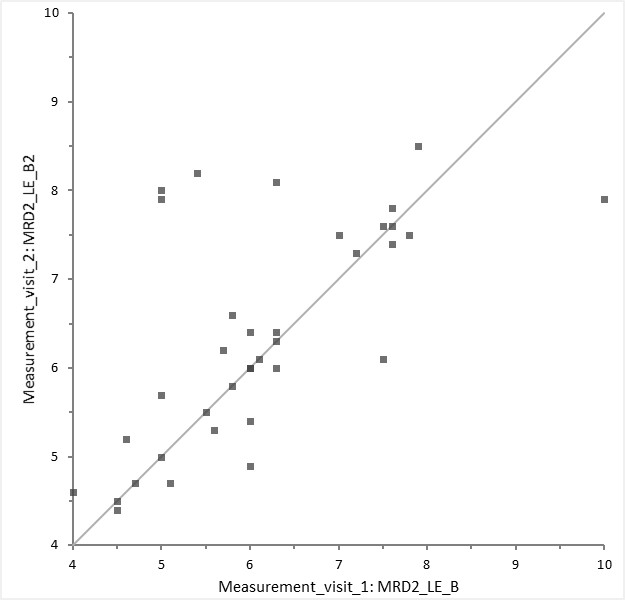


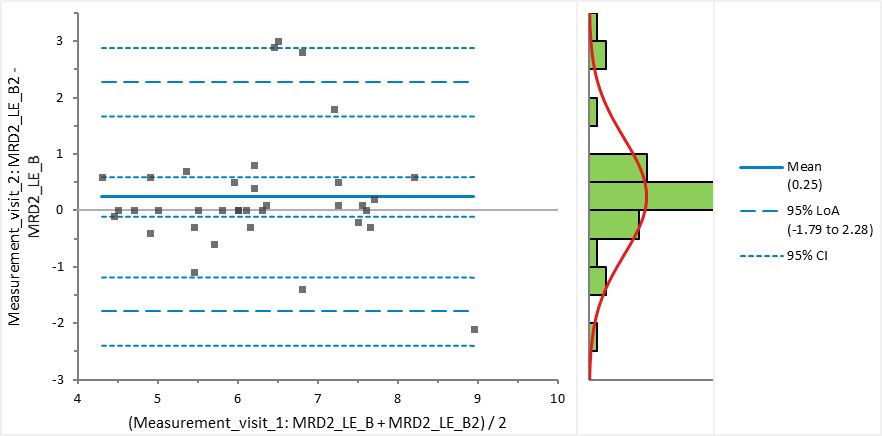


| Parameter | Estimate | 95% CI | | SE |
| --- | --- | --- | --- | --- |
| Mean difference | 0.25 | -0.104 | to 0.598 | 0.173 |
| 95% Lower LoA | -1.79 | -2.391 | to -1.180 | 0.298 |
| 95% Upper LoA | 2.28 | 1.675 | to 2.885 | 0.298 |
